# Strategies to Stabilize Dalbavancin in Aqueous Solutions; Section-2: The Effects of 2 Hydroxypropyl-β-Cyclodextrin and Acetate Buffer with and Without Divalent Metal Ions

**DOI:** 10.3390/pharmaceutics16121503

**Published:** 2024-11-22

**Authors:** Sardar M. Jakaria, David E. Budil, James Murtagh, Graham Revilla

**Affiliations:** 1 Hikma Pharmaceuticals, Bedford, OH 44146, USA; jimmur55@gmail.com; 2 Department of Chemistry and Chemical Biology, Northeastern University, Boston, MA 02115, USA; grahamrevilla@gmail.com

**Keywords:** dalbavancin in aqueous solution, dalbavancin liquid, dalbavancin injection solution, dalbavancin with cyclodextrin, dalbavancin cyclodextrin interaction by ITC and NMR

## Abstract

**Objectives:** The effect of 2-hydroxpropyl-β-cyclodextrin (2HPβCD) with or without divalent metal ions (Ca^2+^, Mg^2+^, and Zn^2+^) on the stability of dalbavancin in acetate buffer was investigated. **Methods:** Dalbavancin recovery from formulations with 2HPβCD and divalent metal ions after four weeks of storage at 5 °C and 55 °C was measured by RP-HPLC and HP-SEC; a longer-term study was carried out over six months at 5 °C, 25 °C, and 40 °C. Binding of 2HPβCD was characterized by isothermal titration calorimetry (ITC) and nuclear magnetic resonance (NMR). **Results:** The stability of the dalbavancin formulations after 4 weeks at 55 °C in 10 mM acetate buffer was significantly improved with 0.6 mM, 5.5 mM, and 55 mM 2HPβCD relative to without 2HPβCD. No further improvement was observed with the addition of any of the divalent cations. Dalbavancin in a 1:10 molar ratio with 2HPβCD was more stable at a concentration of 1 mg/mL than at 20 mg/mL under accelerated conditions at 40 °C for six months. ITC revealed two 2HPβCD binding sites to dalbavancin in 10 mM acetate: one with a 1:1 stoichiometry and thermodynamics consistent with known cyclodextrin–drug interactions, and a second with 0.1:1 stoichiometry, a positive binding enthalpy, and an unusually large entropy of binding. NMR spectroscopy indicates that dalbavancin exhibits aggregation in acetate buffer that is disrupted by 2HPβCD binding. **Conclusions:** 2HPβCD significantly improves the short- and long-term heat stability of dalbavancin in pH 4.5 acetate buffer at and above molar ratios of 1:1. The strong 1:1 binding of 2HPβCD to dalbavancin demonstrated by ITC confirms that this stability is conferred by the formation of a stable complex. This observation, combined with the NMR results, points to the aliphatic hydrocarbon chain of the glycone moiety as the most likely site of 2HPβCD–dalbavancin interaction.

## 1. Introduction

Dalbavancin is a powerful antibiotic against Gram-positive bacteria that is used to treat serious infections, particularly those affecting the skin and soft tissues. It is a second-generation semisynthetic lipoglycopeptide that has been modified by amidation of the carboxy terminal of the peptide with 3-(diethylamino)-1-propylamine. The drug is effective against a range of bacteria including MRSA and several different species of *Streptococcus*, and it is typically given as an intravenous infusion. Recent reviews [1,2,3,4] emphasize the excellent efficacy, half-life, and safety record of dalbavancin, underscoring the need to develop a thermally stable solution formulation of the drug.

**Figure 1:**
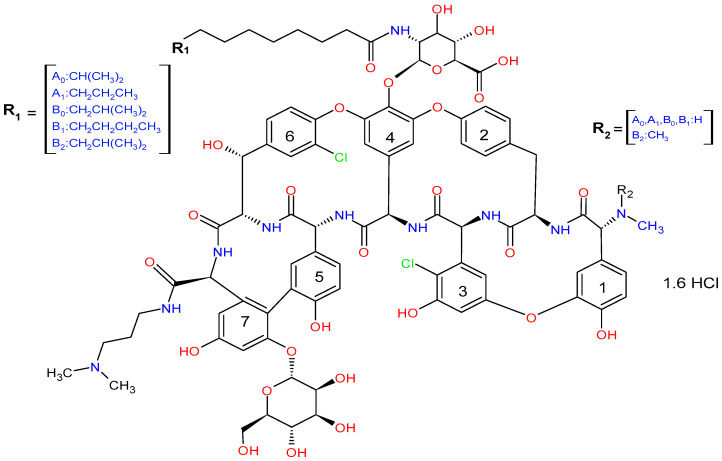
Dalbavancin structural formula [5].

Dalbavancin is a mixture of five similar homologs (A0, A1, B0, B1, and B2), with B0 as the major component [5], all of which are active (Figure 1). They share a common core structure, differing only in the fatty acid side chain of the N-acylaminoglucuronic acid moiety (R1) structure and/or the addition of a methyl group (R2) at the N-terminal amine [5]. The major thermal degradation product of dalbavancin is mannosyl aglycone (MAG), resulting from hydrolysis of the lipophilic glycone tail of the peptide [6]. A recent degradation study has identified several other minor degradation products, including those from acid and alkaline degradation as well as thermal degradation [7].

Using metal ions in combination with a suitable buffer in aqueous solutions has been shown to be an effective way to improve the stability of several peptides [8,9]. The results of our previous study [10] revealed that Ca^2+^ and Zn^2+^ improved dalbavancin’s stability under short-term accelerated conditions at 55 °C for four weeks. However, none of the divalent metal ions studied had a significant effect on dalbavancin’s stability in acetate buffer solutions under accelerated conditions at 40 °C for six months [10].

Another potential excipient to stabilize dalbavancin under such conditions is 2-hydroyxpropyl-β-cyclodextrin (2HPβCD), a cyclic oligosaccharide with attached hydrophobic groups designed to solubilize hydrophobic drug molecules by forming inclusion complexes with them. 2HPβCD is considered safe at relatively high doses and is used in a wide variety of different types of medicines [11,12]. We therefore investigated the effect of 2HPβCD on the stability of dalbavancin in acetate-buffered solutions by screening various formulations of 2HPβCD with and without divalent metal ions.

The purpose of this study was to investigate whether 2HPβCD with or without divalent metal ions can stabilize dalbavancin in aqueous solutions. Stogniew et al. [6] observed that the degradation of dalbavancin strongly depends on the pH of the formulation, with the highest stability observed at pH 4.5. Of the two buffers most commonly used at this pH, citrate produced observable precipitation of dalbavancin, whereas acetate did not [10]. Therefore, all formulations studied in this work were prepared in acetate buffer at pH 4.5.

## 2. Materials and Methods

### 2.1. Materials

All the materials used in this study were commercially available reagents or chemicals. Dalbavancin hydrochloride reference standard and dalbavancin hydrochloride powder were obtained from a supplier with a drug master file. Other chemicals and reagents included 2-hydroxproyl-β-cyclodextrin (2HPβCD), sodium acetate, acetic acid, calcium chloride, magnesium chloride, zinc chloride, potassium chloride, sodium chloride, reagents-plus or ACS grade sodium hydroxide, hydrochloric acid, anhydrous sodium dihydrogen phosphate, HPLC-grade acetonitrile, and type I ultrapure water from a commercially available water purification system. For the NMR work, dalbavancin hydrochloride powder and 2HPβCD were obtained from Millipore Sigma, Burlington, MA, USA.

### 2.2. Formulation and Stability Studies

Dalbavancin was formulated in 10 mM acetate buffer as follows: the buffer was adjusted to pH 4.5 with sodium hydroxide or hydrochloric acid, and 2HPβCD and divalent metal ions (Ca^2+^, Mg^2+^, and Zn^2+^) were added from stock solutions as needed. Dalbavancin was added to a final concentration of either 1.0 or 20 mg/mL, spanning the range of concentrations typically used in clinical settings. The initial dalbavancin concentration was determined by HPLC-UV, monitored at 280 nm. The 2HPβCD solutions were prepared at concentrations of 0.6, 5.5, and 55 mM with or without divalent metal ions in 10 mM acetate buffer. All divalent metal ion solutions (Ca^2+^, Mg^2+^, and Zn^2+^) were prepared from their chloride salts at concentrations of 2, 5, 10, and 50 mM. The prepared solutions were stored in 6R glass type 1 vials for four weeks at 5 °C and 55 °C and protected from light. Based on the results of the screening study at 55 °C for four weeks, dalbavancin formulations of 1 mg/mL and 20 mg/mL in 10 mM acetate buffer (pH 4.5) and 2HPβCD with or without 2, 5, and 10 mM divalent metal salts were selected for longer stability studies over 6 months at 40 °C according to ICH guidelines for long-term and accelerated stability studies for climatic zones III and IV [13]. Sample pH values remained within ±0.2 pH units during both the short-term (4 weeks at 55 °C) and long-term (6 months at 40 °C) accelerated conditions studies.

### 2.3. High-Performance Liquid Chromatography (RP-HPLC and SE-HPLC) Methods

In-house procedures for determining dalbavancin recovery by RP-HPLC and SEC-HPLC have been previously described [10,14] and will be summarized here. A commercially available HPLC instrument equipped with a pump, an autosampler, and a photodiode array detector was used to separate dalbavancin from its degradation products chromatographically. Separation of all compounds was achieved using either a commercially available 250 × 4.6 mm C18 column (5 μm particle size, USP L-1 column packing) or a 300 × 4.6 mM SEC-3 size exclusion column (3 μ particle size) column to determine the amount of monomeric dalbavancin as a fraction of the total remaining dalbavancin. Samples were prepared in a 30%:70% (*v*/*v*) solution of acetonitrile and water for RP-HPLC and a 20%:80% (*v*/*v*) solution for SE-HPLC. The SEC-HPLC chromatography was conducted isocratically using a 20%:80% (*v*/*v*) mixture of acetonitrile with aqueous 150 mM phosphate buffer at pH 7.0. Details of the RP-HPLC solvent program and other experimental conditions for both methods have been given elsewhere [10].

### 2.4. Isothermal Titration Calorimetry (ITC)

Isothermal titration calorimetry (ITC) was used to investigate the interaction between dalbavancin and 2HPβCD in 10 mM acetate buffer at pH 4.5. Microcalorimetric titrations of 2HPβCD to dalbavancin were conducted using a Nano ITC low-volume calorimeter (TA Instruments, New Castle, DE, USA). Solutions of 300 μL of 10 mM dalbavancin in 10 mM acetate pH 4.5 were placed in the sample cell, an equal volume of the same buffer was introduced into the reference cell, and 50 μL of 55 mM 2HPβCD in the same buffer was placed in the calorimeter syringe. After an initial equilibration period of 10 min, automated titrations were conducted at 25 °C up to a 2HPβCD/dalbavancin molar ratio of 2:1. The incremental titrations consisted of 20 injections every 300 s, with an initial injection of 1.5 μL followed by 19 injections of 2.5 μL and a stirring rate of 250 s^−1^. The calorimetric trace was baseline-corrected, and the effective heat of the interaction between the glycopeptide and cyclodextrin determined by integrating the peak observed after each injection. The heat data were corrected for dilution and mixing effects by titrating the 2HPβCD buffer solution into plain buffer. All measurements were performed in triplicate. The data were processed using the Nano Analyze software from TA Instruments and independently checked using equations from reference [15] programmed in MATLAB R2020a Update 4 [16].

### 2.5. Nuclear Magnetic Resonance (NMR) Spectroscopy

NMR was used to investigate possible interactions between dalbavancin and 2HPβCD. ^1^H NMR spectra were recorded on a 400 MHz Bruker (Billerica, MA, USA) Avance Neo NMR spectrometer for 10 mM dalbavancin in 10 mM acetate buffer pH 4.5 doped with 5% (*v*/*v*) D_2_O, both without 2HPβCD and with 2HPβCD present in a 1.2:1 molar ratio to dalbavancin. The water peak from these samples was suppressed using excitation sculpting with gradients (Bruker pulse program zgesgp [17]). For comparison, a ^1^H spectrum of dalbavancin in the solvent DMSO-*d*6 was also recorded.

## 3. Results

### 3.1. Dalbavancin Stability in 2HPβCD and Acetate Buffer Solution

**Figure 2:**
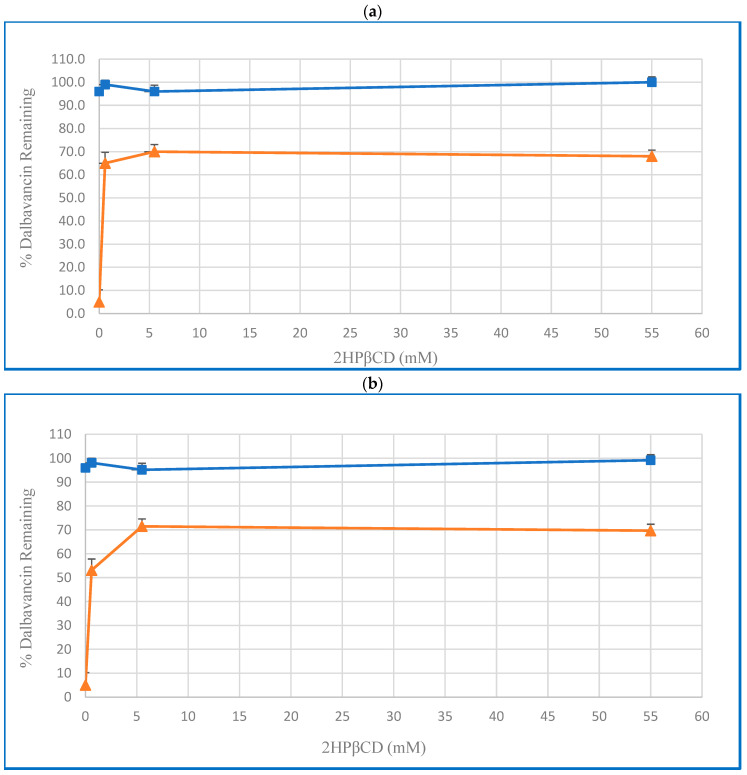
Effect of 2HPβCD concentration on the recovery of 1 mg/mL dalbavancin in 10 mM acetate buffer (pH 4.5) after 4 weeks of storage at 5 °C (■) and 55 °C (▲). (**a**) Dalbavancin recovery determined by RP-HPLC. (**b**) Dalbavancin monomer recovery determined by HP-SEC. The results are depicted as averages of three independent measurements ± SD.

The effect of 2HPβCD on dalbavancin’s stability after four weeks of storage at 5 °C and 55 °C in acetate buffer solution was studied. Samples were prepared with 1 mg/mL (0.55 mM) dalbavancin in 10 mM acetate buffer at pH 4.5 both without 2HPβCD and with 2HPβCD concentrations of 0.6, 5.5, and 55 mM. The recovery of dalbavancin was determined by both RP-HPLC and RP-SEC; the results from the two methods were quite consistent, with the exception of the points at 0.6 mM 2HPβCD at 55 °C. The 2HPβCD significantly increased the stability of the dalbavancin relative to dalbavancin alone under these conditions. In the absence of 2HPβCD, only 5% of the dalbavancin remained, whereas 65%, 70%, and 68% of the dalbavancin remained with 0.6, 5.5, and 55 mM 2HPβCD, respectively, as shown in Figure 2. The most stable solution was 1 mg/mL dalbavancin with 5.5 mM of 2HPβCD in acetate buffer. 

### 3.2. Dalbavancin Stability in Acetate-Buffered Solutions Containing 2HPβCD and Divalent Metal Ions

**Figure 3:**
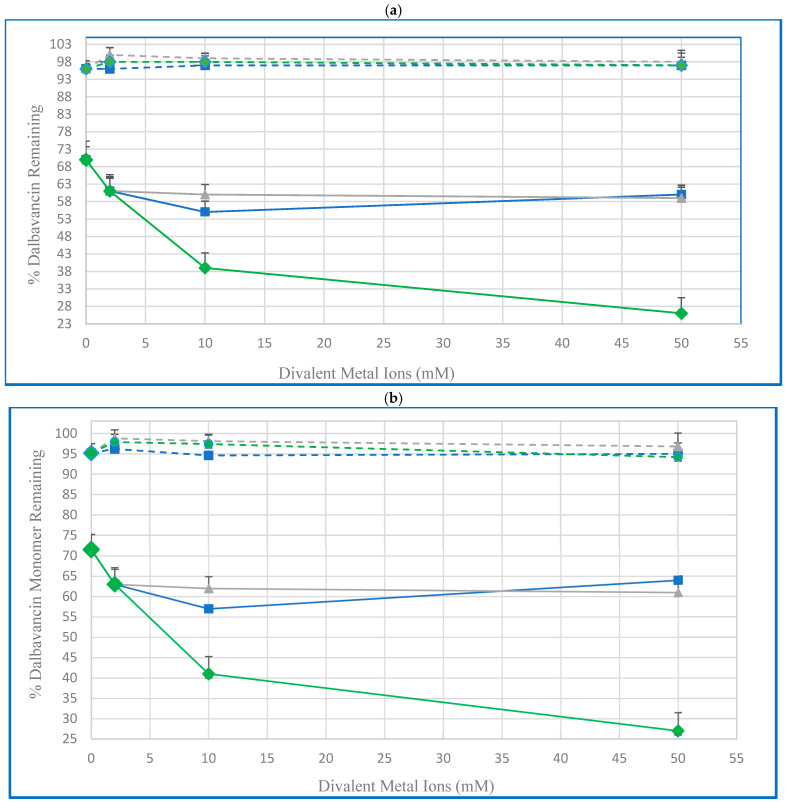
Effect of Ca^2+^, Mg^2+^, and Zn^2+^ concentration on the recovery of dalbavancin (1 mg/mL) in 5.5 mM 2HPβCD and 10 mM acetate buffer (pH 4.5) after 4 weeks of storage with different ions (Ca^2+^ (■), Mg^2+^ (▲), Zn^2+^ (♦) at 5 °C (dashed lines) and 55 °C (solid lines). Dalbavancin recovery determined by (**a**) RP-HPLC and (**b**) HP-SEC.

The additional effect of divalent metal ions (calcium, magnesium, and zinc) on the stability of 1 mg/mL dalbavancin in 10 mM acetate buffer with 5.5 mM 2HPβCD was studied with metal ion concentrations of 0, 2, 5, 10, and 50 mM. Figure 3 plots the recovery of dalbavancin as determined by RP-HPLC and RP-SEC after four weeks of storage at 5 °C and 55 °C as a function of the concentration for each ion. It can be seen from Figure 3 that at 5 °C, the dalbavancin remained stable over the four-week study, with recoveries ≥ 94% for all ions and concentrations studied. It was therefore impossible to discern whether any of the ions had any stabilizing effect. However, at 55 °C, all the ions had an apparent destabilizing effect, reducing the 70% recovery observed with 2HPβCD and zero ions to at most 63% for 2.0 mM Mg^2+^ and Ca^2+^ and as low as 27% for 50 mM Zn^2+^.

### 3.3. Long-Term Stability of Dalbavancin in Selected Formulations Containing Acetate and 2HPβCD with and Without Divalent Metal Ions

Long-term stability studies for six months at 5 °C, 25 °C, and 40 °C were conducted for dalbavancin in 10 mM acetate buffer pH 4.5 in selected formulations, including 2HPβCD with and without Ca^2+^, Mg^2+^, and Zn^2+^. The temperature of 40 °C was chosen to simulate tropical conditions [13,18]. Two dalbavancin concentrations, 1 mg/mL and 20 mg/mL, were studied, and the dalbavancin recovery was determined by RP-HPLC.

At 5 °C and 25 °C, the recoveries exceeded 92% after six months for both dalbavancin concentrations in all formulations studied. The effects of Ca^2+^, Mg^2+^, and Zn^2+^ were tested for different concentrations of dalbavancin and 2HPβCD. As in the short-term studies described above, no significant additional stabilization was observed for any of the ions at any of the ion concentrations studied over the course of six months.

At 40 °C, significant degradation of the dalbavancin was observed after six months. Table 1 summarizes three key results from the studies at this temperature. The first two of the three lines in the table compare the recoveries for the two dalbavancin concentrations at the same molar ratio of 2HPβCD/dalbavancin (10:1). Interestingly, the recovery was appreciably lower at the higher dalbavancin concentration, decreasing from about 80% at a dalbavancin concentration of 1.0 mg/mL to about 70% at 20 mg/mL. The last two of the three lines in the table compare two different 2HPβCD concentrations at the higher dalbavancin concentration, showing nearly identical recoveries for 2HPβCD/dalbavancin ratios of 10:1 and 5:1 at the higher dalbavancin concentration.

**Table 1:** Yield of dalbavancin after six months at 40 °C in 10 mM acetate buffer pH 4.5 for selected formulations.

Dalbavancin Concentration		2HPβCD Concentration	Dalbavancin Recovery
mg/mL	mM	mM	%
1.0	0.55	5.5	79.9
20.0	11	110	69.2
20.0	11	55	70.9

### 3.4. Dalbavancin Binding to 2HPβCD Determined by ITC

**Figure 4:**
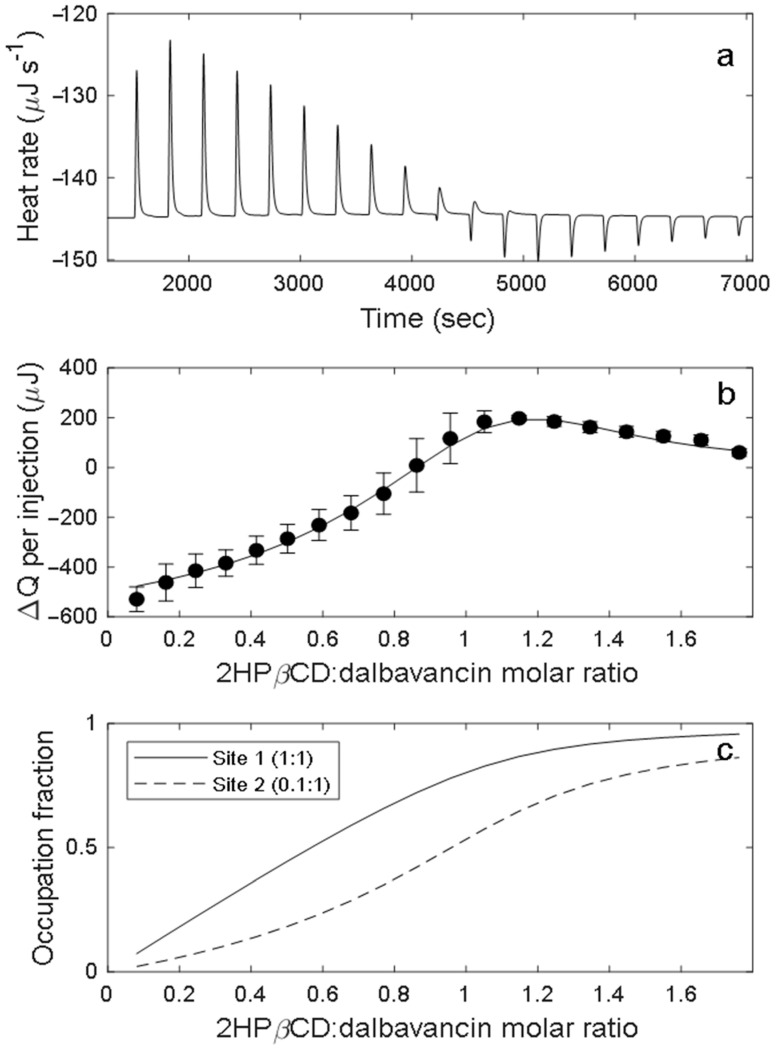
(**a**) Representative ITC calorimetric trace of 55 mM 2HPβCD in 10 mM dalbavancin in sodium acetate buffer at pH 4.5, exhibiting exothermic (positive peaks) and endothermic (negative peaks) binding modes. (**b**) Heat change after each injection obtained by integrating the baseline-corrected peaks of the calorimetric trace. Error bars represent the standard deviation of three independent runs. Solid line represents the best fit of a binding model with two independent binding sites. (**c**) Fractional occupation of the two binding sites vs. 2HPβCD/dalbavancin molar ratio.

The existence of binding interactions between 2HPβCD and dalbavancin was tested by ITC. Figure 4a shows a representative calorimetric trace of an incremental titration of 55 mM 2HPβCD into 10 mM dalbavancin in 10 mM sodium acetate buffer at pH 4.5 and 25 °C. The titration was noticeably biphasic, exhibiting exothermic peaks at the start of the titration and transitioning to a set of endothermic peaks above a 1:1 2HPβCD/dalbavancin molar ratio. The average peak areas for three independent runs after subtracting the peaks from the blank titration are plotted in Figure 4b; error bars indicate the standard deviations.

Given the biphasic nature of the experimental data, only multiple-site models were considered in the analysis. Least-squares minimization of thermodynamic parameters was attempted for two different binding models. The first model assumed two types of independent binding sites with different association constants, binding enthalpies, and numbers of binding sites of that type; the second model assumed sequential binding at two sites with different association constants and binding enthalpies. Satisfactory fits to the data could not be achieved with the sequential binding model.

Table 2 summarizes the parameters for each of two independent sites obtained from fitting the independent-site model. These include the association equilibrium constants $K_{a1}$ and $K_{a2}$, the numbers of each type of site $n_{1}$ and $n_{2}$, and the binding enthalpies $\Delta H_{1}$ and $\Delta H_{2}$. For each site, the free energy of binding was calculated as $\Delta G=-RT\ln K_{b}$, and the entropy of binding was calculated as $\Delta S=(RT\ln K_{a}+\Delta H)/T.\;$The uncertainties reported in Table 1 are the standard deviation from the fits to three separate runs. Figure 4c shows the fractional occupation of Type 1 sites, for which there is one 2HPβCD per dalbavancin molecule, and Type 2 sites, for which there is only 0.1 per dalbavancin, as a function of the 2HPβCD/dalbavancin molar ratio.

### 3.5. NMR Spectroscopy of Dalbavancin and 2HPβCD in Acetate Buffer

**Figure 5:**
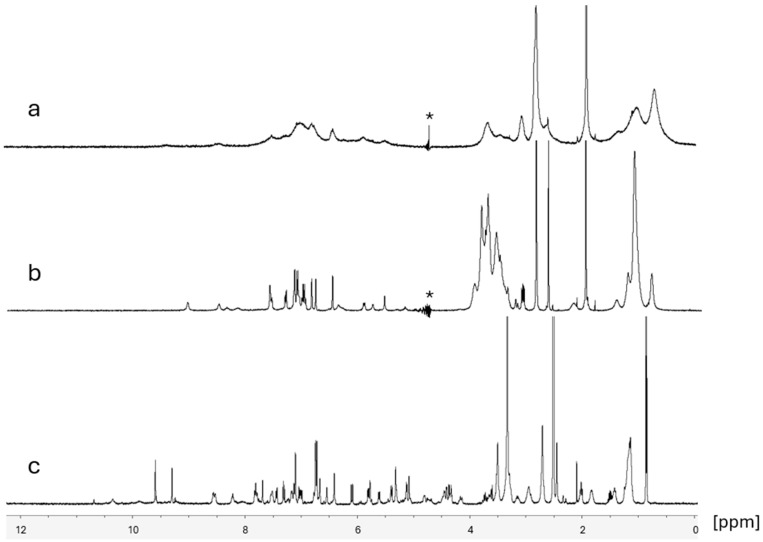
Results of 400 MHz ^1^H NMR spectra of 10 mM dalbavancin in (**a**) 10 mM acetate buffer with 5% D_2_O, (**b**) the same solution with 2HPβCD in a 1.2:1 molar ratio to dalbavancin, and (**c**) in DMSO-*d*6. Asterisks mark the position of the suppressed water peak. The sharp peak at 1.90 ppm in (**a**,**b**) is attributable to the acetate anion.

Figure 5 compares the ^1^H NMR spectra of 10 mM dalbavancin in 10 mM acetate buffer at pH 4.5 without excipients (Figure 5a) and in the presence of 2HPβCD in a 1.2:1 molar ratio of 2HPβCD to dalbavancin (Figure 5b). The ^1^H spectrum of dalbavancin alone was significantly broadened, indicating that the molecule was moving relatively slowly, most likely due to aggregation in the aqueous buffer; however, there was no visual evidence of cloudiness or precipitation in the sample. In the presence of 2HPβCD in the same buffer, most of the peaks sharpened considerably to line widths more typical of solution-state NMR spectra (Figure 5b). This spectrum also exhibited broad features near 1.0 ppm and between 3 and 4 ppm, consistent with published spectra of 2HPβCD in aqueous media [19]. The ^1^H spectrum of dalbavancin in DMSO-*d*6 (Figure 5c) exhibited narrow lines that corresponded closely to the spectrum provided by the supplier.

## 4. Discussion

These studies demonstrate that the stability of dalbavancin in aqueous solution at concentrations used in clinical settings was greatly increased in 10 mM acetate buffer in combination with 2HPβCD. Since the pH of all the formulations studied remained within ±0.2 pH units of 4.5, the pH with the highest dalbavancin stability [6], the pH was not a factor affecting dalbavancin’s stability in the different sample solutions.

In the short-term study for 4 weeks at 55 °C, the recovery of 1 mg/mL dalbavancin was 70% in the presence of 5.5 mM 2HPβCD, representing a 14-fold increase over the recovery from just acetate buffer. Higher molar ratios of 2HPβCD did not produce any further stabilization; in fact, since no recovery measurements were made for 2HPβCD concentrations between 0.6 mM and 5.5 mM, it may be that the optimal stability can be realized at concentrations below 5.5 mM. No further improvement in the dalbavancin recovery was observed with the addition of Ca^2+^, Mg^2+^, or Zn^2+^ salts to the formulation at this temperature. In fact, these ions appeared to reduce the stabilizing effect of 2HPβCD slightly, in contrast to some other peptide-based drugs [20,21]. Although this effect was minor, it may suggest some sort of competition between 2HPβCD and ions for binding to dalbavancin.

The long-term studies at 40 °C confirmed the absence of any effects of divalent metal ions over a six month period, but they did reveal a noticeable reduction in stability when the dalbavancin concentration was increased at a constant 2HPβCD/dalbavancin molar ratio of 10:1. Specifically, the recovery was reduced from about 80% to about 71% upon raising the dalbavancin concentration from 1.0 to 20 mg/mL. This effect may reflect an increased aggregation of the dalbavancin at the higher concentration, either reducing binding to the cyclodextrin or sterically impeding hydrolytic attack at the glycosidic linkage. The long-term results also indicate that reducing the 2HPβCD concentration to 55 mM at the higher dalbavancin concentration did not change the amount recovered, suggesting that the minimal molar ratio needed to achieve optimal stabilization may be less than 5:1.

The ITC results provide strong evidence for direct binding between 2HPβCD and dalbavancin. The presence of both exothermic and endothermic peaks in the ITC experiments clearly shows that this binding occurred in two distinct modes corresponding to two independent sites with different binding constants, occupancy numbers, and binding enthalpies. The first of these sites had a binding constant and exothermic binding enthalpy that fell within the range of values typically observed for cyclodextrin–drug binding [22,23,24], although both were towards the upper end of their respective ranges. The relative magnitude of the binding enthalpy was typical of binding that is primarily due to hydrophobic interactions [22]. This, in combination with the occupancy number of 1.0 for this site, suggests that this binding mode involves a specific molecular interaction between dalbavancin and 2HPβCD.

In contrast, the second binding interaction between 2HPβCD and dalbavancin was quite atypical of cyclodextrin–drug binding. A positive binding enthalpy is extremely rare for this type of interaction and always small in magnitude [22,23,24]. While positive entropy changes are more commonly observed in cyclodextrin–drug binding, the +220 J K^−1^ mol^−1^ determined for the second dalbavancin binding mode was well above the magnitude typically seen for such interactions. These observations and the very low occupancy number for the second site suggest a much less specific interaction between 2HPβCD and dalbavancin that may involve a substantial disruption of the peptide core conformation that produces a relatively disordered structure. Thus, the large positive enthalpy could reflect the breaking of hydrophobic interactions or hydrogen bonds that maintain the most stable structure of dalbavancin, while the binding equilibrium is driven by the large increase in the entropy of the resulting complex. These observations suggest that 2HPβCD/dalbavancin molar ratios much greater than 2:1 may destabilize dalbavancin at higher temperatures, consistent with kinetic studies [7].

An obvious candidate for the 1:1 binding site of 2HPβCD is the hydrophobic lipid chain of the drug molecule, which could form a complex with the hydrophobic propanol side chains of the cyclodextrin. This is consistent with the typical mode of action of cyclodextrins, in which they form an inclusion complex with all or part of the drug. It is also consistent with the observation that 2HPβCD solubilizes dalbavancin in aqueous buffer near pH 7.0, where dalbavancin by itself is uncharged and insoluble [14]. By analogy with other vancomycin-group antibiotics [25], dalbavancin presumably aggregates via hydrophobic interactions between the lipid chains that are disrupted by complexation with 2HPβCD.

The broad NMR lines from dalbavancin in aqueous buffer provide additional evidence that dalbavancin aggregates in aqueous solution to slow its apparent rotational motion, even at pH 4.5. The lines sharpened in the presence of 2HPβCD in water, indicating faster rotation of the dalbavancin and suggesting that the cyclodextrin increased the mobility of the drug molecule by disrupting the aggregation. The observed narrow lines of dalbavancin in DMSO demonstrate a similar increase in mobility by solubilization.

Aggregation of the dalbavancin may also explain the discrepancy between the yields measured using reverse-phase and size exclusion columns at 0.6 mM 2HPβCD (Figure 2). At this relatively low 2HPβCD concentration, there may have been residual aggregation, such that aggregates were separated from monomeric dalbavancin in the size exclusion column, reducing the apparent yield of dalbavancin.

The observed stabilizing effect of 2HPβCD is consistent with its binding to the lipid moiety of dalbavancin. The major degradation pathway of dalbavancin in aqueous solution is hydrolysis of the glycosidic linkage between the lipid and the glycopeptide to form mannosyl aglycone (MAG) [6]. Hydrolytic attack at this linkage could therefore be at least partially blocked by the binding of a large group to the lipid chain of the glycone. Finally, the absence of any additional stabilizing effect from divalent cations is consistent with a binding interaction that is dominated by hydrophobic interactions.

Although there have been no previous reported studies of the possible stabilizing effect of 2HPβCD on dalbavancin, cyclodextrins have been observed to suppress hydrolysis of glycosidic linkages in smaller compounds. Uekama et al. [26] observed that β cyclodextrin significantly suppressed the acid hydrolysis of digoxin, a cardiac glycoside, via the formation of an inclusion complex. K Miyake et al. studied the improvement of the solubility and oral bioavailability of rutin by cyclodextrins [27] and found lower rates of alkaline hydrolysis of rutin to produce quercetin, again through the formation of a relatively stable inclusion complex [27,28]. Although the 2HPβCD cavity is not large enough to accommodate the entire dalbavancin molecule, these analogous cases do illustrate how cyclodextrins can sterically block hydrolytic attack at the glycoside linkage.

## 5. Conclusions

This study demonstrates that the stability of dalbavancin in aqueous solution could be significantly increased by 2HPβCD in acetate buffer both at 55 °C and 40 °C. However, no further stabilization could be achieved with the addition of divalent cations, in contrast to other peptide-based drugs. The stability was also reduced appreciably at the higher dalbavancin concentration studied, suggesting there may be some optimal concentration of the drug in formulation. Isothermal titration calorimetry (ITC) revealed a specific 1:1 binding of the 2HPβCD to the drug. In combination with solubility and NMR data, the results point to a complex between the cyclodextrin and the lipid tail of dalbavancin. Such a complex could inhibit the hydrolytic cleavage of the glycosidic bond connecting the lipid tail to the glycopeptide body, which forms mannosyl aglycone (MAG) as the major degradation product. These findings represent a major step towards developing heat-stable formulations of dalbavancin suitable for distribution to patient populations without access to a cold chain [29].

## Figures and Tables

**Table 2:** Least-squares parameters from a model of two independent binding sites to the ITC data.

Parameter	Value
$K_{a1}\;(M^{-1})$	$\left(4.4\pm 0.9\right)\times 10^{3}$
$n_{1}$	1.04±0.07
$\Delta H_{1}\;(kJ\;{mol}^{-1})$	−5.0±0.3
$\Delta G_{1}\;(kJ\;{mol}^{-1})$	−20.8±0.5
$\Delta S_{1}\;(J\;K^{-1}\;{mol}^{-1})$	52.9±0.7
$T\Delta S_{1}\;(kJ\;{mol}^{-1})$	15.8±0.2
$K_{a2}\;(M^{-1})$	$\left(1.2\pm 0.2\right)\times 10^{3}$
$n_{2}$	0.102±0.004
$\Delta H_{2}\;(kJ\;{mol}^{-1})$	46±4
$\Delta G_{2}\;(kJ\;{mol}^{-1})$	−17.6±0.4
$\Delta S_{2}\;(J\;K^{-1}\;{mol}^{-1})$	220±11
$T\Delta S_{2}\;(kJ\;{mol}^{-1})$	66±3

## Data Availability

The original contributions presented in this study are included in the article. Further inquiries can be directed to the corresponding authors.
